# Pangenome Analysis Reveals Novel Contact-Dependent Growth Inhibition System and Phenazine Biosynthesis Operons in *Proteus mirabilis* BL95 That Are Located in An Integrative and Conjugative Element

**DOI:** 10.3390/microorganisms12071321

**Published:** 2024-06-28

**Authors:** Andrey Tatarenkov, Iván Muñoz-Gutiérrez, Isabel Vargas, Judith Behnsen, Luis Mota-Bravo

**Affiliations:** 1 School of Biological Sciences, University of California, Irvine, CA 92697, USA; tatarenk@uci.edu (A.T.); imunozgu@uci.edu (I.M.-G.); isabel.vargas@pennmedicine.upenn.edu (I.V.); 2 Department of Microbiology and Immunology, University of Illinois Chicago, Chicago, IL 60612, USA; jbehnsen@uic.edu

**Keywords:** integrative conjugative element (ICE), bacterial toxins, bacterial competition, contact-dependent growth inhibition (CDI) system, phenazine biosynthesis, phylogeny of *Proteus mirabilis*, *Enterobacteriaceae*

## Abstract

*Proteus mirabilis* is a leading cause of urinary tract infections and a common commensal of the gastrointestinal tract. Our recent study (JB) showed that *P. mirabilis* strain BL95 employs a novel contact-dependent killing system against enteric bacteria in the mouse gut and in vitro. To uncover the genetic determinants of this system, we performed whole-genome sequencing of BL95 and compared it with 98 complete genomes of *P. mirabilis*. BL95 carries 56 coding sequences (CDSs) not found in other *P. mirabilis*. Over half of these unique genes are located on a novel integrative conjugative element (ICE) named ICE*Pm2*, inserted in tRNA-Phe and exclusive to BL95. ICE*Pm2* has integration, conjugation, and DNA replication modules nearly identical to ICE*Pm1* (common in *P. mirabilis*), but ICE*Pm2* of BL95 carries two unique operons for *P. mirabilis*—a phenazine biosynthesis and a contact-dependent growth inhibition (CDI) system. ICE*Pm2* is absent in the *P. mirabilis* (AR_0156) closest to BL95 and it is present in the genomes of several *Escherichia coli* from mouse intestines, indicating its recent horizontal mobilization. BL95 shares over 100 genes of five different secretion systems with other *P. mirabilis*, mostly poorly studied, making a large pool of candidate genes for the contact-dependent growth inhibition.

## 1. Introduction

*Proteus mirabilis* is a gram-negative bacterium best known for swarming motility and urease production. This bacterium is a leading causative agent of catheter-associated urinary tract infections, a common commensal of the gastrointestinal tract, and also widely distributed in soil, stagnant water, and sewage [1,2].

Colonization of the gastrointestinal tract is polymicrobial, and *P. mirabilis* possesses several systems to outcompete other microorganisms, including distinct strains of its own species [2]. *P. mirabilis* commonly competes with unrelated conspecific strains using the contact-dependent type VI secretion system (T6SS), which results in Dienes lines when two different strains swarm and encounter one another on an agar plate [3,4,5]. To outcompete other bacterial species during colonization, the genome of *P. mirabilis* encodes seventeen fimbrial operons and thirteen fimbrial orphan genes, as well as genes involved in the assembly of the type IV pili [6]. Additionally, the genome of *P. mirabilis* encodes six potential autotransporters of the type V secretion system (T5SS), half of which appear to be adhesins and the other half are potential toxins [6]. The genome also contains genes encoding two-partner secretion systems (TPS) of T5SS that act in a contact-dependent manner [7]; one of these is the HpmB-HpmA system having hemolysin activity and another is the contact-dependent growth inhibition system (CDI; CdiA/CdiB) employed by some gram-negative bacteria to outcompete co-residing bacteria [8,9]. *P. mirabilis* can also outcompete co-occurring bacteria through the secretion of ammonia and other volatile compounds [10].

The entire set of genes from all representatives of a bacterial species is known as its pangenome [11]. Each isolate of a bacterial species carries a core set of genes ensuring species’ existence, which can be supplemented by accessory genes that confer fitness to a specific niche [12]. The accessory genes are often localized in genomic islands, such as integrative and conjugative elements (ICEs), which are exchanged by horizontal gene transfer between bacteria. ICEs are integrated into a host genome, but they carry genetic apparatus allowing them to excise, produce conjugation machinery, and transfer to other strains and species, moving accessory genes with them [13].

ICE*Pm1* is an ICE that is common in *P. mirabilis*, reaching 100% frequency in urinary isolates and 65% in isolates from other body parts [14]. Nearly identical genomic islands were reported in other bacterial species, such as *Providencia stuartii* and *Morganella morganii*. The 92.6 kb ICE*Pm1* from *P. mirabilis* HI4320 has a highly modular structure typical for ICEs, including integration, conjugation, and replication core modules, as well as interspersed modules for accessory genes such as ones encoding an iron acquisition system and membrane proteins [14]. ICE*Pm1* integrates into the tRNA-Phe gene, forming 52 bp direct repeats (DR) as flanking regions. *P. mirabilis* has two copies of tRNA-Phe; experiments by Flannery et al. [12] showed that ICE*Pm1* preferentially integrates into the second copy of this gene, called tRNA-PheV, but can also integrate into another copy, called tRNA-PheU.

Recently, an isolate of *P. mirabilis* BL95 isolated from the feces of a lab mouse (*Mus musculus* strain C57BL/6) was reported to employ a contact-dependent killing system against coexistent *Enterobacteriaceae* [15]. Several physiological characteristics pointed out that such a system may be novel for *Proteus* and, possibly, for Enterobacterales. However, no attempts have been made yet to find its genetic underpinning. The objective of our study was to sequence, annotate, and characterize the genome of *P. mirabilis* BL95, identify unique features of its genome, especially those involved in killing or growth suppression of competing bacteria, and to conduct a comparative analysis of a novel integrative conjugative element (ICE) harboring genes that confer competitive advantage.

## 2. Materials and Methods

### 2.1. Library Construction, Sequencing, and Assembly

The whole genome of *P. mirabilis* BL95 was sequenced using Illumina and Oxford nanopore approaches. Detailed information about methods used can be found in [16]. Briefly, bacteria were grown in 2 mL of tryptic soy broth for 18 h at 35 °C and shaking at 200 rpm and genomic DNA was extracted and purified using phenol/chloroform [17]; after first extraction with phenol/chloroform, RNAse A was added to the sample and incubated at 37 °C for 15 min. DNA quantity and quality were assessed using measurements with Nanodrop (ThermoFisher, Waltham, MA, USA), Qubit (Invitrogen, Carlsbad, CA, USA), and visualization on 0.8% agarose gel. One genomic library was prepared following the Nextera DNA Flex Library Prep protocol (Illumina, San Diego, CA, USA). The library was sequenced on an Illumina MiniSeq sequencer using a 150-bp paired-end sequencing approach. FASTQ file generation pipelines included an adapter trimming option. A total of 628.3 Mb of raw data was generated from 4,160,654 trimmed reads. An Oxford Nanopore Technologies (ONT) (Oxford, UK) library was prepared using SQK-LSK109 and EXP-NBD196 (using the manufacturer’s protocol, without the optional fragmentation step), loaded to a FLO-MIN106 flow cell, and run in a MinION device (Oxford Nanopore Technologies plc, Oxford, UK). In total, 27,847 ONT reads were generated with an average length of 10,517 bp. By using Unicycler v0.4.8-beta [18] with default settings, both sequencing datasets were assembled into a complete circular chromosome.

### 2.2. Annotation and Identification of Genomic Features

Different annotation methods result in distinct annotations, mainly with regards to naming genetic features, but also in identification of the features themselves. To ensure thorough annotation and gene identification, we used pgap [19], Prokka [20], and BV-BRC Annotation service [21]. We used pgap annotation when referring to specific genetic features by their locus_tag, in order to be consistent with accessions deposited in GenBank. Integrative elements (such as ICE and IME) were identified using ICEFinder in ICEberg 2.0 [22]. Protein secretion systems were identified with TXSScan [23]. Orphan genes of the contact-dependent growth inhibition system (CDI) encoding effector toxins (*cdiA-CT*) and immunity proteins (*cdiI*) were identified by searching for GenBank accessions where these genes are a part of the complete CDI system (*cdiB/cdiA/cdiI*). Naming of the orphan genes followed a system used by Poole et al. [24], by adding the suffix ‘o’ and the index corresponding to the order position of the orphan after complete CDI. Promoters and terminators were predicted with BPROM [25] and ARNold [26] software, respectively, using the default parameters.

### 2.3. Genomic Comparative Analyses

The Proksee server (https://proksee.ca, accessed on 16 December 2022) was used to generate the annotated map of the strain BL95 chromosome [27]. Similarities between genomes and individual genomic features were shown using EasyFig [28]. Geneious Prime 2022.1.1 (https://www.geneious.com, accessed on 30 June 2023) was used for sequence visualization, navigation, and alignment.

In our comparative genomic analyses, we considered 98 isolates of *P. mirabilis* (not counting *P. mirabilis* BL95) with complete genomes from the RefSeq database (Table 1). We did not include partially assembled genomes because we wanted to be sure about the location of genomic features (chromosome vs. plasmid), and relative position of such features in the genome. We also included representatives of three closely related species (*P. vulgaris*, *P. hauseri*, and *P. penneri*) as outgroups for phylogeny reconstruction. Table 1 lists GenBank and RefSeq assembly accessions, species, strains, and location of ICE*Pm*.

The evolutionary tree of *P. mirabilis* was inferred with concatenated genes of the core genome using Prokka [20] and Roary [29]. First, genomes of all *P. mirabilis* were annotated by Prokka. Then, homologous protein-encoding genes were identified by similarity and a matrix of genes presence/absence was constructed using Roary. To be considered homologous, we used a threshold of 80% at amino acid level. Finally, genes present in all *Proteus* strains were individually aligned and concatenated using Roary. Altogether, 2449 genes were individually aligned and then concatenated, for a total of 2,301,814 nucleotide positions in the final dataset. This dataset was used to reconstruct the phylogenetic tree in MegaX [30] using the Neighbor-Joining method with Kimura-2-parameter distance and the bootstrap test (100 replicates).

The matrix of genes presence/absence described above was used to identify genes unique to *P. mirabilis* BL95.

## 3. Results

### 3.1. Phylogenetic Analysis

**Figure 1:**
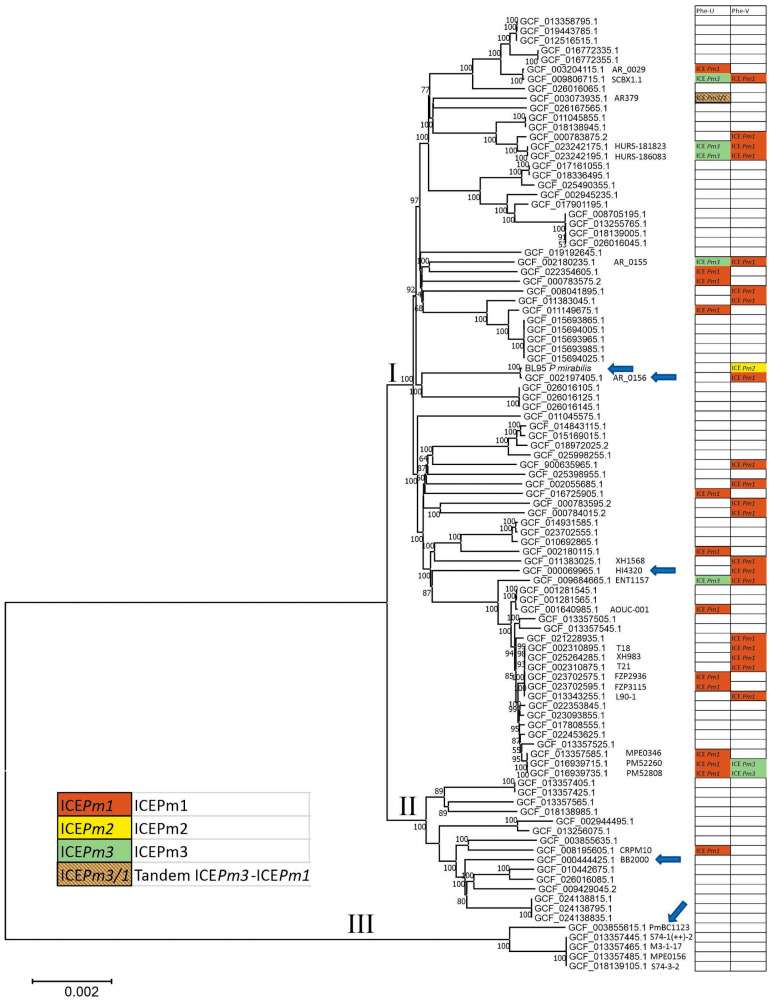
Phylogeny of *P. mirabilis*. Isolates are named after RefSeq Assembly Accession. Additionally, isolates mentioned in the text have strain name shown. Roman numerals above branches designate three major clades. The chart on the right shows type (ICE*Pm1*, ICE*Pm2*, and ICE*Pm3*), distribution among isolates, and insertion locus (tRNA-PheU or tRNA-PheV) of integrative and conjugative elements. Arrows point to isolates used for the genome comparison in Figure 2.

To better understand the origin of the genomic features of *P. mirabilis* BL95, we constructed a core-genome phylogeny of *P. mirabilis* strains from the RefSeq database using all complete genomes (N = 99, including BL95; Table 1). Evolutionary relationships of *P. mirabilis* based on 2449 genes shared by all 99 isolates are shown in Figure 1. Three well-delineated clades can be distinguished on the tree (shown by Roman numerals above branches). BL95 belongs to the largest clade I, which includes *P. mirabilis* HI4320 (NCBI RefSeq assembly GCF_000069965.1), often used in experiments and a reference genome for *P. mirabilis* [6]. The nucleotide identity between BL95 and HI4320 across the core genome is 0.99452. The second clade is represented by strain BB2000, a model organism for self-recognition [31]. The nucleotide identity between BL95 and BB2000 across the core genome is 0.99289. Finally, the third, basal clade III, is made up of five isolates obtained from feces or digestive tract of animals. The nucleotide identity between BL95 and PmBC1123 from clade III is 0.97325.

The closest relative to BL95 is *P. mirabilis* AR_0156 of unknown origin. AR_0156 and BL95 differ at only 189 nucleotide positions across 2449 core genome genes, resulting in a nucleotide identity of 0.99991 and indicating that these strains share a very recent common ancestor. Assuming mutation rate equals to 10^−9^—or one substitution in one billion bases per generation—we estimate that the common ancestor of BL95 and AR_0156 lived 42,954 bacterial generations ago, or just about five years ago, if the generation time is one hour. Altogether, *P. mirabilis* forms a very compact group, well delineated from other *Proteus* species and exhibiting high nucleotide identity (>97%) even between its most diverged representatives.

### 3.2. Pangenome Analysis and Comparative Genomics

Due to extensive horizontal gene transfer in bacteria, even the most closely related bacteria may have drastically different phenotypes, if they acquired distinct mobile genetic elements (such as plasmids, ICEs, and transposons). To have a comprehensive view of intraspecies variation, it is thus important to consider the species pangenome, which includes core genome as well as accessory genes. Supplementary Figure S1 summarizes the pangenome of *P. mirabilis*. While the core genome is made up of 2449 genes, members of this species carry a total of 11,364 genes. Furthermore, more than half of the genes, 7017, can be found in less than 15% of strains. This suggests that despite genetic cohesiveness at the core genome, *P. mirabilis* is a very polymorphic species due to the presence and absence of accessory genes.

**Figure 2:**
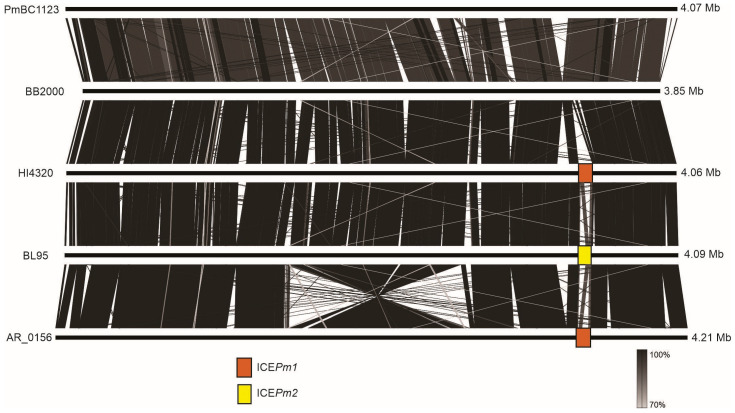
Alignment of chromosomes in representative *P. mirabilis*. Included are BL95, its closest sister taxon, AR_0156, and representatives of the three major clades shown in Figure 1. Gray rectangles indicate regions of homology, and their shades show sequence similarity according to the vertical scale bar. Colored rectangles indicate ICE*Pm1* and ICE*Pm2*. Comparison was performed using EasyFig [28].

Genomic rearrangements are another potential source of variation. Figure 2 shows the alignment of chromosomes from five representative isolates of *P. mirabilis*. Overall, the alignment reveals a high degree of synteny (noted previously in [2]). Even strain PmBC1123 from the diverged clade III shows a similar order of genes as most other strains. On the other hand, the strain AR_0156 closely related to BL95, has a large inversion that encompasses 25% of its chromosome. Furthermore, several insertions and deletions are interspersed along the chromosomes in all compared strains, including BL95 and AR_0156. This demonstrates that genomic rearrangements, as well as accumulation and loss of genes, driven largely by mobile genetic elements, can happen on a short evolutionary scale and do not correlate with the number of single nucleotide substitutions.

### 3.3. General Features of BL95 Genome

The fully assembled genome of BL95 consists of a 4,086,891 bp-long chromosome. No plasmids were found. The chromosome contains 3749 genes, of which 109 are RNA genes and 3640 are coding sequences (CDSs). Among CDSs, 3595 are complete coding genes and 45 are pseudogenes (frameshifted, incomplete, or containing an internal stop). Among RNA genes, 22 are rRNA genes, 83 are tRNAs, and 4 are ncRNAs. The GC content of the complete genome is 39.2%. The GC content of the core genome (2449 protein coding genes for a total length of 2,293,671 bp) is 40.7%. A schematic representation of the *P. mirabilis* BL95 chromosome is shown in Supplementary Figure S2.

### 3.4. Unique Features of BL95 Genome

Several genomes of *P. mirabilis* have been described [31,32,33] with the reference genome of strain HI4320 providing a detailed description of the genomic features typical for this species [6]. Therefore, in our study we focused on genes and genomic features that are unique to BL95. As shown in Table 2, *P. mirabilis* BL95 carries 56 unique genes (i.e., they are not found in any of the 98 *P. mirabilis* strains).

### 3.5. BL95 Carries a Novel ICE, ICEPm2

ICEFinder identified in BL95 an integrative and conjugative element with modules of accessory genes previously unknown in *P. mirabilis* and not described formally in other species. Below we provide a description of this ICE, which we named ICE*Pm2*, including its structure and key differences from related and well-studied ICE*Pm1*. Furthermore, our comparative bioinformatic analysis uncovered yet another ICE, also related to ICE*Pm1*, which we named ICE*Pm3*.

### 3.6. Structure of ICEPm2

Over half of the genes unique to BL95 are found within a region identified by ICEFinder as an integrative and conjugative element (nucleotides 3,419,106–3,507,006; Figure S2). This 87.9 kb-long ICE is integrated into the 3′ end of the tRNA-PheV, flanked by nearly identical 52 bp-long direct repeats, and carries genes encoding integrase, relaxase, T4SS, and genes involved in DNA replication. A total of 51 bp of the direct repeats overlap the tRNA-PheV gene. The direct repeat at the left-most end (*attL*, nucleotides 3,419,106–3,419,157) has one nucleotide difference from the direct repeat at the right-most end (*attR*, nucleotides 3,506,955–3,507,006). The GC content of this ICE is 50.4%, which is noticeably higher than the GC content of the BL95 genome (39.2%), suggesting that this region was acquired through horizontal gene transfer.

**Figure 3:**
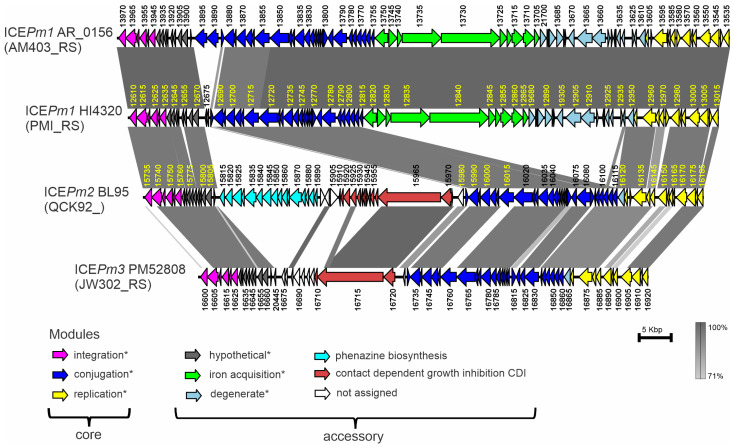
Gene organization of ICE*Pm1*, ICE*Pm2*, and ICE*Pm3* from representative strains of *P. mirabilis* and their comparison using Easyfig [28]. Genes are colored with different colors according to the module to which they belong. Modules marked with asterisks are after [14], the rest are according to the present study. Genes are labeled using locus number of the locus tags; unique for each gene locus number is shown next to gene, while locus name, unique for GenBank accession, is shown in parentheses on the left (e.g., the full locus tag for the first gene in ICE*Pm1* from strain AR_0156 is AM403_RS13970). Complete lists of locus tags are shown in Supplementary Table S1. Homologous regions are outlined by gray rectangles, with their shades showing similarity according to the vertical scale bar.

**Figure 4:**
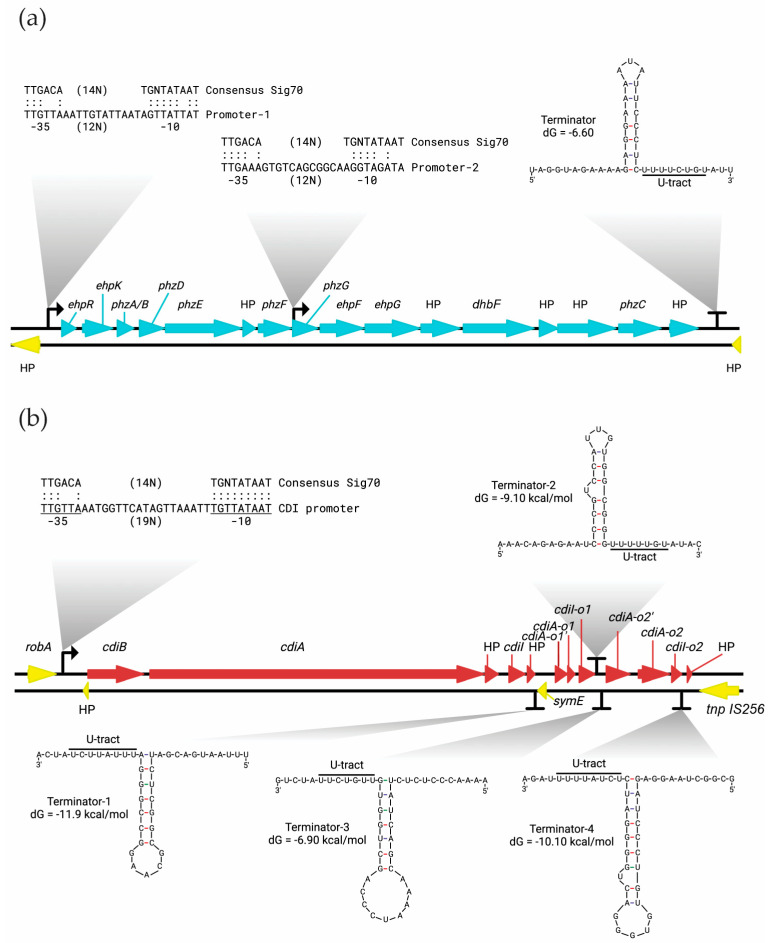
Genetic context of *P. mirabilis* BL95 phenazine (**a**) and CDI (**b**) operons. BPROM and ARNold software predicted the σ^70^ promoters and the terminators respectively, using the default parameters. The −35 and −10 promoter regions are underlined. Every match to the σ^70^ promoter consensus is indicated by a colon (:). The U-tract of the terminators is underlined, and the free energy of the stem-loop regions (dG) is shown next to the stem. (**a**) The genes predicted to be part of the phenazine operon are shown in cyan. The putative σ^70^ promoter-1 is 292 nucleotides upstream of *ehpR*, and the putative σ^70^ promoter-2 is 16 nucleotides downstream of *phzG*’s start codon. The putative terminator is 374 nucleotides downstream of the last hypothetical protein gene that belongs to the operon. (**b**) The genes predicted to be part of the CDI operon are shown in brown. The putative σ^70^ promoter is 642 nucleotides upstream of *cdiB*. The positions of the putative terminators are as follows: the terminator-1 is six nucleotides downstream of *symE*, the terminator-2 is next to the stop codon of *cdiI-o1*, the terminator-3 is 90 nucleotides downstream of *cdiI-o1*, and the terminator-4 is 15 nucleotides downstream of *cdiI-o2*.

Integration in a tRNA-Phe is a hallmark of ICE*Pm1*, an element initially observed in the first sequenced genome of *P. mirabilis* HI4320 [6] and analyzed in detail by Flannery et al. [12,14]. A comparison of ICE*Pm1* and ICE of BL95 showed that they share large blocks of similarity yet have extensive non-shared regions (Figure 3). Considering these substantial differences (see below), from now on, we will refer to this ICE as ICE*Pm2*, a novel ICE in BL95 related to ICE*Pm1*. Flannery et al. [14] distinguished three modules in ICE*Pm1*—integration, conjugation, and DNA replication—that are necessary for its self-transmissibility. Inserted between them are variable modules carrying accessory genes. Our comparative analysis shows that the integration, conjugation, and DNA replication modules of ICE*Pm1* have corresponding matches in ICE*Pm2*. In contrast, the variable modules differ greatly. ICE*Pm2* carries a large block of genes, inserted between the integration and conjugation modules, that is absent in ICE*Pm1* (Figure 3). This block in BL95 ICE*Pm2* carries unique genes for *P. mirabilis* that constitute (1) a novel 16 kb phenazine biosynthesis operon (Figure 4a), and (2) an 18.1 kb contact-dependent growth inhibition system operon (Figure 4b). At the same time, the variable modules of ICE*Pm1* (iron acquisition system and degenerate region), located between its conjugation and DNA replication module, are absent in ICE*Pm2*.

### 3.7. Distribution of ICEPm1, ICEPm2, and ICEPm3 among P. mirabilis Isolates and Variation in Insertion Sites, tRNA-PheV or tRNA-PheU

To find out the distribution of ICEs similar to ICE*Pm1* and ICE*Pm2*, to determine their integration sites, and to characterize the main variants of these elements, we conducted a systematic search of complete *P. mirabilis* genomes (Table 1), as well as other bacteria. Criteria for the search were (1) the identification of the characteristic direct repeats formed by integration into tRNA-Phe, and (2) the presence of modules encoding self-transferability. Of the 99 isolates of *P. mirabilis*, 35 carried ICEs satisfying these criteria, and some of these isolates had two ICE copies. Comparison of these elements showed that in addition to ICE*Pm1* and ICE*Pm2*, there is another ICE, sufficiently diverged to classify it under a different name as ICE*Pm3* (Figure 3). Like ICE*Pm2*, ICE*Pm3* lacks the variable modules of ICE*Pm1* and carries genes encoding CDI; unlike ICE*Pm2*, it lacks the module for phenazine biosynthesis. Although ICE*Pm2* and ICE*Pm3* have more accessory genes in common, ICE*Pm2* has higher genetic similarity to ICE*Pm1* (92.45% ± 0.14%) than to ICE*Pm3* (89.26% ± 0.17%) in shared genes of the integration, conjugation, and DNA replication modules (Figure 3).

ICE*Pm1* is found in 34 isolates; in 20 isolates it is integrated into the tRNA-PheV, and in 14 isolates into the tRNA-PheU (Figure 1). ICE*Pm2* is integrated into the tRNA-PheV and it is found only in BL95. ICE*Pm3* is found in eight isolates, and all these isolates also have ICE*Pm1*; in two isolates, ICE*Pm3* is integrated into the tRNA-PheV, and in six isolates into the tRNA-PheU. When an isolate has simultaneously two ICEs (ICE*Pm1* and ICE*Pm3*), they are integrated in different copies of the tRNA-Phe, with the exception of strain AR379, where both ICEs are associated with the tRNA-PheU (Figure 1). Specifically, ICE*Pm3* is integrated into the tRNA-PheU, while ICE*Pm1* is integrated into the direct repeat right (DR *attR*) of ICE*Pm3*, thus forming a tandem repeat of two ICEs.

Inspection of the distribution of ICE*Pm1*, *-2*, and *-3* on the phylogenetic tree shows that these ICEs have inserted independently multiple times (Figure 1). Furthermore, the frequency of insertions must be high, because closely related BL95 and AR_0156 carry different ICEs (ICE*Pm2* and ICE*Pm1*, respectively), in the same tRNA-PheV locus. Another pair of closely related isolates, AR_0029 and SCBX1.1, carry ICE*Pm1*, which are integrated in different tRNA-Phe loci (U or V), indicating independent insertion. A similar situation is observed in a cluster of six nearly genetically identical isolates (T18, XH983, T21, FZP2936, FZP3115, L90-1), where ICE*Pm1* is integrated in different tRNA-Phe loci. These observations strongly suggest that ICE*Pm1* and ICE*Pm2* are currently active elements.

Identical copies of ICE*Pm3* are found in sister-taxa PM52260 and PM52808, but absent in the closely related MPE0346 and in more distantly related strains (Figure 1), indicating a recent integration. The integration likely occurred in the common ancestor of PM52260 and PM52808 after it separated from MPE0346. Moreover, highly similar copies of ICE*Pm3* (100% coverage and 99.99% identity) are found in six distantly related strains of *Proteus* (AR379, HURS-181823, HURS-186083, PM52260, PM52808, and AR_0155), and furthermore, integrated in different loci (tRNA-PheU or tRNA-PheV). These observations strongly suggest that ICE*Pm3* is an active ICE moving between *P. mirabilis* isolates.

Additional evidence that ICE*Pm1* and ICE*Pm2* are active comes from the pattern of distribution and high identity of these ICEs in other bacteria. For example, ICE*Pm2* in BL95 is nearly identical (100% coverage, 99.91% identity) to such elements in *E. coli* from mice feces (Accessions CP010221.1, CP010206.1, CP010196.1, CP010186.1, CP010213.1). Highly similar copies of ICE*Pm1* across the full length (Coverage: ≥95%; Identity: >99%) were found in over 40 g-negative species, including *Providencia*, *Escherichia*, *Klebsiella*, and *Morganella*. ICE*Pm3* was not found in other bacteria, suggesting that its movement is limited intra-specifically to *P. mirabilis*. Considering high similarity between the eight copies of ICE*Pm3*, it may have formed recently, and it did not have time to spread to other bacteria or was not yet found due to low frequency.

### 3.8. Novel Phenazine Biosynthesis and Contact-Dependent Growth Inhibition System Operons in ICEPm2 in P. mirabilis BL95

#### 3.8.1. Phenazine Biosynthesis Gene Operon in ICEPm2

Embedded in ICE*Pm2,* region 3,431,156–3,446,405 (QCK92_15815–QCK92_15890) of the BL95 chromosome encompasses a cluster of 16 genes involved in phenazine biosynthesis (Figure 4a; Table 3). Six genes in this cluster, *phzA/BCDEFG*, encode the core phenazine biosynthesis enzymes [34,35], gene *ephR* encodes resistance to phenazine, and the remaining genes encode enzymes involved in the modification of phenazine tricycles [35]. The canonical *phzC* gene encodes a 3-deoxy-d-arabino-heptulosonate-7-phosphate (DAHP) synthase, which catalyzes the first step of the shikimate pathway and redirects the intermediates from primary metabolism into phenazine biosynthesis [36]. Although BL95 lacks the DAPH synthase gene, it carries a gene encoding a 3-deoxy-7-phosphoheptulonate synthase which likely performs an analogous function by providing metabolic precursors for the shikimic acid pathway and thus aiding in the synthesis of phenazines [34].

According to BPROM software [25], the genes of the phenazine cluster are regulated by two σ^70^ promoters, one upstream of *ehpR* and another within *phzG* (Figure 4a). The second promoter has probably evolved to increase the transcription level of downstream genes, including *phzC* that encodes for a core phenazine biosynthesis enzyme. The transcription of the operon is terminated by a Rho-independent terminator as predicted by the ARNold software [26] (Figure 4a).

Even though our results indicate that this operon is found in other bacteria, this is the first report of a phenazine operon in *P. mirabilis*. This operon is present with the same genes and in the same order in five isolates of *E. coli* from mice feces (see above, similarity > 99.9%) and two isolates of *P. stuartii* (CP114580.1, CP114582.1, similarity = 94.8%). Additionally, all the genes of the operon but one (encoding 4′-phosphopantetheinyl transferase) are found on a plasmid of *E. coli* (CP076707.1, similarity = 95.5%) and on a chromosome of *Serratia fontinicola* DSM 4576 (CP011254.1, similarity = 76.4%). The high sequence divergence of the *S. fontinicola* phenazine operon from the rest suggests that this cluster existed in such configuration for a considerable time, and it is likely preserved by natural selection. Leise [35] classified this phenazine cluster as type 2, out of six types distinguished in Enterobacterales, and found that this type was associated with ICE integrated in tRNA-Phe, consistent with our finding of this cluster integrated in tRNA-Phe by ICE*Pm2*.

Phenazines are key contributors to many aspects of the biology of their producers [37]. They exhibit broad antimicrobial properties, making their producers more competitive, increasing virulence and playing a role in biofilm formation and iron acquisition [34,35,38,39,40,41].

#### 3.8.2. Contact-Dependent Growth Inhibition Gene Cluster in ICEPm2

Contact-dependent growth inhibition (CDI) system, first described in *E. coli* EC93, is encoded by a locus of three genes, *cdiB*, *cdiA*, and *cdiI* [42]. The CdiB-CdiA proteins form a two-partner secretion system: CdiB is an outer membrane protein that presents the CdiA exoprotein on the cell surface [9,24]. The N-terminal part of CdiA is a delivery system, while the polymorphic C-terminal region (CdiA-CT) is a toxin exhibiting a distinct growth inhibition activity on the target bacteria [7,24,43]. The N- and C- termini are demarcated by the VENN motif. CdiI is an immunity protein that binds to cognate CdiA-CT and blocks its activity, thereby protecting the cell from autoinhibition [24]. A comparative genomic analysis showed that many CDI systems contain “orphan” *cdiA-CT* units not connected to the *cdiA* N-terminus. Usually, a *cdiA-CT* unit is linked to a cognate *cdiI* gene downstream, forming a pair. These pairs are mobile, and can fuse to N-terminus, producing a system with novel inhibition activity [24,43].

**Figure 5:**
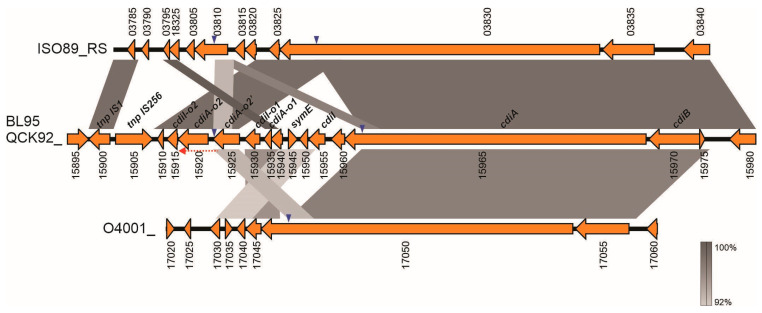
Gene alignment of CDIs to identify orphan gene pairs *cdiA-CT/cdiI* in the CDI region from ICE*Pm2* in *P. mirabilis*. Orphans were identified by finding homologous regions in the full *cdiB/cdiA/cdiI* loci in selected bacterial species. Homologous regions are outlined by gray rectangles, with their shades showing similarity according to the vertical scale bar. CDI regions are labeled according to locus name: ISO89_RS, *M. morganii* 1810122035 (GenBank accession no. NZ_JADICD010000001); QCK92_, *P. mirabilis* BL95 (CP122400); O4001_, *P. stuartii* 2021CK-01196 (CP114580). Genetic features are labeled by locus number; additionally, gene names are shown for BL95. Probable orphan genes are indicated by the suffix ‘-o’. Blue triangles indicate VENN motif. Orphans ‘-o1’ are homologous to *cdiI* and C-terminus of *cdiA* in *P. stuartii*, but region of similarity does not include the VENN motif, and thus these orphans are likely non-functional. Orphans ‘-o2’ are homologous to *cdiI* and C-terminus of *cdiA* in *M. morganii*. The red dotted arrow shows the alternative extent of ORF for *cdiA-o2*, which overlaps the VENN motif. Comparison was performed using EasyFig [28].

Our comparative genomic analysis showed that a cluster of genes that belong to the CDI system is found in the ICE*Pm2* of *P. mirabilis* strain BL95, almost immediately upstream of the phenazine biosynthesis operon (Figure 3 and Figure 4b). The CDI system (nucleotides 3449845–3467528, loci QCK92_15910–QCK92_15970) and the phenazine operon are separated by transposases of the IS1 and IS256 families. The CDI system of BL95 has the three central structural genes that form a CDI system, namely *cdiB*, *cdiA*, and *cdiI* (Figure 5). Additionally, as shown in Figure 4b, the CDI system of BL95 has two “orphan” *cdiA-CT/cdiI* sets that are indicated with the suffixes ‘-o1’ and ‘-o2’. Moreover, our results showed that most of CDI ORFs (except *cdiA* and *cdiB*) are found only in BL95 and no other *P. mirabilis* strains.

An alignment between ICE*Pm2* and ICE*Pm3* showed that the *cdiB* genes in both ICEs are closely related homologs. Additionally, both ICEs share a closely related *cdiA* homolog; however, the 3′-ends of *cdiA*, encoding C-terminus, are non-homologous (Figure 3). The *cdiA* gene in the ICE*Pm2* is followed by “orphan” *cdiA-CT/cdiI* sets that are absent in other *P. mirabilis*. Some of the genes in these orphan sets have similarity to other genes in the BL95 CDI cluster. For example, locus QCK92_15955 encoding CdiI is 70.1% similar to locus QCK92_15930 encoding the “orphan” CdiI-o1. Additionally, an 830 bp-long portion of *cdiA* (QCK92_15965) is 95.2% similar to the “orphan” *cdiA-o2* (QCK92_15925). Interestingly, one gene in the middle of the CDI cluster in ICE*Pm2* does not seem to be part of the CDI system but rather represents another toxin system of the SymE family (QCK92_15945).

The genes of the BL95 CDI cluster have numerous correspondences to GenBank sequences, with those most similar ones shown in Figure 5 and Figure S3. The strain BL95 CDI cluster is almost 100% identical to the CDI of *E. coli* M19 isolated from mice feces (GenBank Accession CP010221). Additionally, a shorter region with high identity is found in another *E. coli* Mt1B1 (CP028714). It is important to notice that annotations of the CDI system are lacking in these accessions (Figure S3).

Comparison of the BL95 CDI with the *P. stuartii* strain 2021CK-01196 (CP114580.1) and *M. morganii* 1810122035 CDIs reveals several matches, which give some insights on how this cluster may have formed in strain BL95. As shown in Figure 5, all these bacteria harbor *cdiB* and *cdiA* with an identity higher than 92%. However, the similarity of *cdiA* only extends to the VENN motif (i.e., *cdiA-CT* are different) indicated by blue arrowheads in Figure 5. Interestingly, the *cdiA-CT* and cognate *cdiI* of *P. stuartii* are found in BL95, but relative to *P. stuartii* they translocated a few hundred base pairs downstream of *cdiA*, forming orphans *cdiA-o1* and *cdiI-o1*. These results indicate that the *cdiA* in BL95 arose from a progenitor similar to that of *P. stuartii* by the replacement of *cdiA-CT* and cognate *cdiI* with new variants. The displaced *cdiA-CT* and cognate *cdiI* were not lost but became “orphans”.

Similarly, the C-terminus of *cdiA* and cognate *cdiI* of *M. morganii* formed the “orphans” *cdiA-o2* and *cdiI-o2* in *P. mirabilis* BL95 (Figure 5). Moreover, the *cdiA-o2′* (QCK92_15925) and the intergenic region upstream of *cdiA-o2* share high similarity with *M. morganii*, suggesting that in the past, *cdiA-o2* and *cdiA-o2′* formed a single long ORF that was split into separate ORFs by a single nucleotide insertion (Figure 5).

According to BPROM [25], the *cdiB*, *cdiA*, and *cdiI* are regulated from a single σ^70^ promoter (Figure 4b), forming an operon. The σ^70^ promoter perfectly matches the −10-consensus sequence (TATAAT), including the −10-extended (TGN). The ARNold software [26] revealed that the transcription termination for these genes could be achieved by the terminator-2 (Figure 4b). The software predicted three more terminators in the antisense strand that prevent a transcriptional read-through from the antisense strand (Figure 4b). For example, terminator-1 stops the *symE* gene transcription and prevents any interference with the CDI operon transcription. It is important to mention that the U-tract next to the stem-loop dictates the directionality of the terminators [44].

### 3.9. Abundance of Secretion Systems in P. mirabilis BL95

A search of the strain BL95 genome with TXSScan resulted in a list of over 100 genes that belong to secretion systems type I, III, IV, V, and VI (Supplementary Table S2). Of these, T3SS is known to be used by bacteria to kill eukaryotic cells [8,15], whereas all other systems are potential candidates for interbacterial competition.

In addition to the CdiB/CdiA system, the secretion systems of strain BL95 include other two-partner systems (TPS). One TPS of T5bSS in nucleotides position 1,198,117–1,205,409 (loci QCK92_05550–QCK92_05560; ShlB/DUF637, T5bSS) is distantly related to the *cdiB/A/I* of ICE*Pm2* and is likely involved in competition with other bacteria [6]. The locus encoding that system is not associated with mobile elements, it is found in all *P. mirabilis* and other members of the genus, and it is likely intrinsic to the genus. Another TPS of T5bSS, called *hpmB/hpmA*, is in nucleotides position 2,840,233–2,846,685 (loci QCK92_13275 and QCK92_13280). This is a well-known system from *P. mirabilis* described in 1990 that encodes cytolysin/hemolysin with cytotoxic activity [7,45].

## 4. Discussion

An earlier study by one of us [15] observed that a commensal *P. mirabilis* strain BL95 outcompeted commensal *E. coli* in the gut of neonatal mice and killed *E. coli* and other enterobacteria in vitro. Experiments showed that killing required direct contact of *P. mirabilis* and the competing bacteria. Furthermore, *P. mirabilis* BL95 demonstrated unique features of inhibition, compared to other functionally characterized contact-dependent growth inhibition systems, such as the CDI in *E. coli* [42]. One such feature was that BL95 developed inhibitory properties when reaching the stationary phase when grown in liquid culture, and not in the exponential phase [42]. Another distinguishing feature was high inhibitory effectiveness in shaking liquid culture, in addition to being effective on solid media [15]. Importantly, the ability to kill enterobacteria extended to other *P. mirabilis* strains [15], although it is not known if the same systems were involved in the competition by these strains as they were not functionally characterized to the same detail as BL95.

As a first step in identification of candidate genes encoding this contact-dependent inhibition system with novel functional properties, we sequenced and characterized the genome of strain BL95 and compared it with other *P. mirabilis* with complete genomes. Placing BL95 in a larger phylogenetic framework is a promising approach to narrow down potential candidates. For example, if a CDI with certain properties is scattered non-systematically across the phylogeny, this would indicate that the CDI genes are located on a mobile genetic element, such as a genomic island or a plasmid, prompting the identification of this element to find the genes involved. Alternatively, the CDI may be restricted to a group of related *Proteus* (clade), suggesting the need to find the common genomic locus present in this clade but not in other *Proteus* isolates.

Our phylogeny represents the first attempt to characterize the diversity of *P. mirabilis* using complete genomes and to systematically study the distribution of ICE*Pm* in the species. Overall, *P. mirabilis* forms a tight-knit genetic group with nucleotide identity higher than 97% between the most diverged members. It is also well separated from the closest species of *Proteus*, making the species assignment unambiguous. *P. mirabilis* BL95 belongs to the largest clade, out of three clades, that also includes reference strain HI4320. Despite high similarity of the core genome, *P. mirabilis* has a vast suite of accessory, or cloud genes, that are found in a subset of strains or even in single strains. For example, *P. mirabilis* BL95 possesses 56 genes not found in other strains of *P. mirabilis*. It should be made clear that not all these genes bestow unique functions or features to BL95. Some of these genes may be diverged duplicates of genes present in other isolates and have similar functions. Still, even if the general function is similar (e.g., contact-dependent growth inhibition), the genetic variation might account for unique fine-tuning that may be crucial in some environments (e.g., expanding the range of target species or conferring an ability to establish cell contact in liquid media). On the other hand, some genes are unique to *P. mirabilis* BL95, such as the phenazine biosynthesis operon present in ICE*Pm2*. However, the functions of the majority of unique genes in strain BL95 are yet unknown.

Phenazines exhibit broad antimicrobial properties by generating toxic reactive oxygen species and helping their producers to outcompete other bacteria in their ecological niche [36]. The broad-spectrum antimicrobials can be particularly beneficial for species occupying a niche with diverse microbiota, so that different potential competitors are targeted [46]. Furthermore, by acting as electron shuttles, phenazines help aerobic bacteria maintain redox homeostasis in low-oxygen environments through their ability to mediate the reoxidation of NADH under oxygen-limiting conditions, explaining the importance of phenazine production for biofilm formation, as mature biofilms are often hypoxic [34,40]. *P. mirabilis* is notorious for forming biofilms in catheters, being a leading cause of the catheter-associated urinary tract infections [1,2]. Phenazines also regulate diverse physiological functions, serving as intercellular signals and regulators of gene expression [40].

Our analysis revealed the presence of genes potentially encoding multiple secretion systems. Many of these systems have homologies to the reference strain HI4320 or other *P. mirabilis*. Although a few of these systems were characterized previously [2], the exact function for most of them is not well studied, leaving multiple candidates for contact-dependent killing described by Kiani et al. [15]. For instance, by using a mutant *tssM* of BB2000 which lacks a functional T6SS, Kiani et al. [15] ruled out T6SS as a candidate system involved in contact-dependent growth inhibition exhibited by BL95 and other *P. mirabilis* strains. However, the *tssM* mutation affected only one of T6SS, the same one known to be involved in the contact-dependent competition between strains of *Proteus*. Other T6SS found in BL95 and other *Proteus* may have different functions, e.g., they can be involved in interspecies bacteria competition. Pertinent, a genome-wide transposon mutagenesis study of *P. mirabilis* showed that the known *P. mirabilis* T6SS operons were overrepresented as fitness factors during coinfection with *P. stuartii*, indicating a potential role for T6SS in mediating competitive and cooperative interactions during polymicrobial infection [47].

One of the most common ICEs in *P. mirabilis* is ICE*Pm1* [14]. In strain HI4320 this element consists of six modules, three of which are the core backbone modules [48] involved in self-replication and self-transmission, and the other three are accessory gene modules. Surprisingly, strain BL95 has an element with the same core backbone modules, but distinct accessory genes modules. Specifically, ICE of BL95 lacked the iron acquisition and degenerate modules of ICE*Pm1* (located between conjugation and replication modules) but carried the phenazine biosynthesis and CDI modules (located between integration and conjugation modules). Considering the extent of structural and functional changes, we propose that the ICE of BL95 be recognized as a different element which we named ICE*Pm2*. Furthermore, our bioinformatic analysis also showed that the mobilome of *P. mirabilis* possesses yet another ICE with the same core backbone but distinct accessory genes, which we named ICE*Pm3*. ICE*Pm2* and ICE*Pm3* are similar, but the latter lacks the phenazine biosynthesis operon. Furthermore, the *cdiA* genes of ICE*Pm2* and ICE*Pm3* have different *cdiA-CT* termini, and orphan toxin/immunity gene pairs of the CDI module are unique to ICE*Pm2* (and BL95).

Another argument for considering ICE*Pm1*, ICE*Pm2* and ICE*Pm3* as distinct ICEs is their pattern of presence in some strains. Flannery et al. [12] noted that only one ICE*Pm1* copy can be present in any single bacterium (inserted in either tRNA-pheU or -pheV). Our analyses showed that several strains carry ICEs in both tRNA-Phe, but in all such strains the inserted ICEs are of different types (ICE*Pm1* and ICE*Pm3*), and never are they of the same type. All three ICEs have essentially the same core backbone modules determining their integration. Therefore, the lack of strains carrying simultaneously ICEs of the same type is likely not because such integration does not occur, but rather due to evolutionary pressure removing such combinations. Possession of an ICE might implicate both advantages (new functions due to accessory genes) and disadvantages (e.g., metabolic burden to replicate the ICE) [13]. ICEs of different types carry distinct accessory modules that confer distinct advantageous properties, whereas possession of two copies of the same ICE is redundant, leading to the elimination of strains with such combination. We found one strain, *P. mirabilis* AR379, that had ICE*Pm1* and ICE*Pm3* tandemly arranged in tRNA-pheU, which shows that accumulation of ICEs by accretion may be a way for bacteria to create a larger, more complex mobile element [49]. Accumulation by accretion is common for many composite transposons, such as those bound by IS*26* [50], but such mechanism is less known for ICEs [51,52]. Some authors proposed that accumulation by accretion may be an important mechanism to increase ICEs diversity [13,53].

ICE*Pm1-2-3* are somewhat unusual among ICEs because they can integrate into either tRNA-Phe locus. Wozniak and Waldor [49] report that most ICEs targeting tRNA loci integrate into only one of a few tRNA loci, with only two known exceptions, ICE*clc* and PAPI-1, that can integrate into either tRNA locus. ICE*Pm1-2-3* not only can integrate into either locus, but they can be found in both loci simultaneously and even integrate into the same locus repeatedly by accretion, forming tandems.

One complication for the study of CDI systems is the lack of specialized software or databases for their identification in a genome. Annotating genes encoding contact-dependent inhibition systems is not trivial, because of their similarity to other better-characterized TPS, such as hemolysins and hemagglutinins. As a result, *cdiA* genes are often annotated as hemagglutinins, and *cdiB* genes as ShlB/FhaC/HecB hemolysins [54]. However, CdiB/CdiA pairs constitute a distinct subfamily of the two- partner secretion proteins [55]. CdiA is a large filamentous protein whose N-terminal region is homologous to that of *Bordetella pertussis* filamentous hemagglutinin (FHA). Indeed, the N-terminal regions of CdiA and FHA contain the typical hemagglutinin repeats and a hemagglutination activity domain [55]. In contrast to FHA, the CdiA N-terminal region ends with a conserved domain designated PT-VENN, helping its identification.

The *cdiB* genes can be correctly identified by their proximity to and association with *cdiA*, as well as by sequence comparison with representatives of TPS subfamilies. In our study, locus QCK92_15970 was incorrectly annotated by the NCBI as a ‘ShlB/FhaC/HecB family hemolysin secretion/activation protein CDS’, but our phylogeny using proteins from UniProt showed that it is most closely related to CdiB2 from *Burkholderia pseudomallei*. The position of QCK92_15970 immediately upstream of *cdiA* containing VENN motif further confirms that this gene should be annotated as *cdiB*, rather than ShlB/FhaC/HecB.

Annotating orphan genes is even more challenging because orphan *cdi-CT*s are short ORFs that do not start with methionine initiation codon. Moreover, *cdi-CT* and cognate *cdiI* genes are highly variable and little studied [43]. As a result, these orphan genes are either annotated as hypothetical, or they are not detected by annotation software. We also observed that different annotation software can suggest different ORFs in the same region, with some ORFs corresponding to actual ‘orphan’ genes and others being spurious. One way to unambiguously identify orphan genes is to find them as part of a complete CDI system (*cdiB/cdiA/cdiI*). Using this approach, we identified orphan genes in the CDI cluster of ICE*Pm2* of strain BL95. Specifically, one pair of BL95 orphans was found in the complete CDI system of *P. stuartii*, and another pair in the complete CDI of *M. morganii* (Figure 5).

Comparison of CDIs in *P. mirabilis* BL95 and *M. morganii* also demonstrated how orphan genes are shaped in the course of evolution (Figure 5). This comparison shows that in the past *cdiA-o2* and *cdiA-o2’* formed one continuous ORF, perhaps making a longer orphan *cdiA-CT-o2*. Single nucleotide insertion interrupted this ancestral ORF, resulting in two ORFs. One of these ORFs became a shorter orphan encoding toxin (*cdiA-o2*), while the other ORF is likely a non-functional pseudogene (*cdiA-o2’*), because it does not have C-terminus and thus it cannot be used for displacing C-termini in *cdiA* genes to produce new toxin variants. As noted previously for T6SS [56], understanding the importance of orphan genes in bacterial competition will require their careful detection, curation, and systematic analysis. It is essential to create databases of CDI genes, including orphan genes, similar to ISFinder that identifies insertion sequences [57] or services of the CGE (https://www.genomicepidemiology.org, accessed on 1 April 2024).

Previous work established that *P. mirabilis* BL95 outcompetes co-occurring enterobacteria in vivo and in vitro in a contact-dependent manner. Here we sequenced and analyzed the fully assembled genome of BL95 and showed that it carries (1) a phenazine biosynthesis operon unique to *P. mirabilis* and (2) a contact-dependent growth inhibition system operon, previously not reported in this species. These operons are adjacent to each other and embedded in an ICE that we named ICE*Pm2*. Future studies will establish whether the phenazine and/or CDI operons in ICE*Pm2* are responsible for the competitive features displayed by strain BL95. It may be significant that ICE*Pm2* carrying CDI operon originates from *P. mirabilis* isolated from mice intestines. Gastrointestinal (GI) tract has a rich and competitive microbiota, compared to the urinary tract which normally harbors a smaller number of microorganisms (mainly *Lactobacillus* and other Firmicutes) [58]. The presence of ICE*Pm2* with the CDI operon in BL95 may be connected to the highly diverse microenvironment of the GI tract. Some previous studies suggested that CDI systems are particularly effective when direct cell-cell contact between competing strains is common giving an advantage to their hosts [9,59]. Indicative, that other bacterial species carrying ICE*Pm2* were also of GI origin. ICE*Pm2* is a novel ICE that shares core modules—integration, conjugation, and replication—with ICE*Pm1*, but carries distinct accessory modules. Our bioinformatic analyses of *P. mirabilis* isolates from the GenBank revealed yet another previously undescribed element named ICE*Pm3*, similar to ICE*Pm2*, but lacking the phenazine biosynthesis operon. The distribution of ICE*Pm1-2-3* on the phylogeny of *P. mirabilis* shows their multiple independent integration in different *Proteus* lineages and indicates that these elements are currently active. Notably, ICE*Pm1* and ICE*Pm3* can be found simultaneously in the same strain, but none of them (and ICE*Pm2*) are found duplicated. One interpretation of this observation is that these elements co-exist when they confer distinct advantages due to non-overlapping accessory modules; however, isolates containing duplicate copies of the same ICE are selected against due to redundancy and metabolic burden.

## Figures and Tables

**Table 1:** Ninety-nine *P. mirabilis* strains with complete genomes used in the phylogeny reconstruction and comparative analyses. Three additional species of *Proteus* were used as outgroups.

RefSeq Assembly Accession	GenBank Accession	Species	Strain	Insertion Locus of ICE*Pm1*	Insertion Locus of ICE*Pm2*	Insertion Locus of ICE*Pm3*
BL95_P_mirabilis	CP122400	*Proteus mirabilis*	BL95		tRNA-PheV	
GCF_000069965.1	NC_010554.1	*Proteus mirabilis*	HI4320	tRNA-PheV		
GCF_000444425.1	NC_022000.1	*Proteus mirabilis*	BB2000			
GCF_000783575.2	NZ_CP026062.1	*Proteus mirabilis*	FDAARGOS_81	tRNA-PheU		
GCF_000783595.2	NZ_CP026059.1	*Proteus mirabilis*	FDAARGOS_80	tRNA-PheV		
GCF_000783875.2	NZ_CP026051.1	*Proteus mirabilis*	FDAARGOS_67	tRNA-PheV		
GCF_000784015.2	NZ_CP026044.1	*Proteus mirabilis*	FDAARGOS_60	tRNA-PheV		
GCF_001281545.1	NZ_CP012674.1	*Proteus mirabilis*	CYPM1			
GCF_001281565.1	NZ_CP012675.1	*Proteus mirabilis*	CYPV1			
GCF_001640985.1	NZ_CP015347.1	*Proteus mirabilis*	AOUC-001	tRNA-PheU		
GCF_002055685.1	NZ_CP020052.1	*Proteus mirabilis*	AR_0059	tRNA-PheV		
GCF_002180115.1	NZ_CP021550.1	*Proteus mirabilis*	AR_0159	tRNA-PheU		
GCF_002180235.1	NZ_CP021694.1	*Proteus mirabilis*	AR_0155	tRNA-PheV		tRNA-PheU
GCF_002197405.1	NZ_CP021852.1	*Proteus mirabilis*	AR_0156	tRNA-PheV		
GCF_002310875.1	NZ_CP017082.1	*Proteus mirabilis*	T21	tRNA-PheV		
GCF_002310895.1	NZ_CP017085.1	*Proteus mirabilis*	T18	tRNA-PheV		
GCF_002944495.1	NZ_CP026571.1	*Proteus mirabilis*	BC11-24			
GCF_002945235.1	NZ_CP026581.1	*Proteus mirabilis*	GN2			
GCF_003073935.1	NZ_CP029133.1	*Proteus mirabilis*	AR379	tRNA-PheU		tRNA-PheU
GCF_003204115.1	NZ_CP029725.1	*Proteus mirabilis*	AR_0029	tRNA-PheU		
GCF_003855615.1	NZ_CP034091.1	*Proteus mirabilis*	PmBC1123			
GCF_003855635.1	NZ_CP034090.1	*Proteus mirabilis*	PmSC1111			
GCF_008041895.1	NZ_CP042907.1	*Proteus mirabilis*	VAC	tRNA-PheV		
GCF_008195605.1	NZ_CP043332.1	*Proteus mirabilis*	CRPM10	tRNA-PheU		
GCF_008705195.1	NZ_CP044028.1	*Proteus mirabilis*	K817			
GCF_009429045.2	NZ_CP045538.2	*Proteus mirabilis*	CRE14IB			
GCF_009684665.1	NZ_CP044136.1	*Proteus mirabilis*	ENT1157	tRNA-PheV		tRNA-PheU
GCF_009806715.1	NZ_CP047112.1	*Proteus mirabilis*	SCBX1.1	tRNA-PheV		tRNA-PheU
GCF_010442675.1	NZ_CP048404.1	*Proteus mirabilis*	N18-00201			
GCF_010692865.1	NZ_CP048787.1	*Proteus mirabilis*	CC15031			
GCF_011045575.1	NZ_CP042857.1	*Proteus mirabilis*	1701092			
GCF_011045855.1	NZ_CP047352.1	*Proteus mirabilis*	ZA25			
GCF_011149675.1	NZ_CP049753.1	*Proteus mirabilis*	PmBR607	tRNA-PheU		
GCF_011383025.1	NZ_CP049941.1	*Proteus mirabilis*	XH1568	tRNA-PheV		
GCF_011383045.1	NZ_CP049942.1	*Proteus mirabilis*	XH1569	tRNA-PheV		
GCF_012516515.1	NZ_CP051260.1	*Proteus mirabilis*	STP3			
GCF_013255765.1	NZ_CP053894.1	*Proteus mirabilis*	JPM24			
GCF_013256075.1	NZ_CP053898.1	*Proteus mirabilis*	YPM35			
GCF_013343255.1	NZ_CP045257.1	*Proteus mirabilis*	L90-1	tRNA-PheV		
GCF_013357405.1	NZ_CP053615.1	*Proteus mirabilis*	MPE0734			
GCF_013357425.1	NZ_CP053616.1	*Proteus mirabilis*	MPE0767			
GCF_013357445.1	NZ_CP053614.1	*Proteus mirabilis*	S74-1(++)-2			
GCF_013357465.1	NZ_CP053681.1	*Proteus mirabilis*	M3-1-17			
GCF_013357485.1	NZ_CP053682.1	*Proteus mirabilis*	MPE0156			
GCF_013357505.1	NZ_CP053683.1	*Proteus mirabilis*	MPE0027			
GCF_013357525.1	NZ_CP053718.1	*Proteus mirabilis*	MPE4069			
GCF_013357545.1	NZ_CP053684.1	*Proteus mirabilis*	MPE5139			
GCF_013357565.1	NZ_CP053685.1	*Proteus mirabilis*	MPE5203			
GCF_013357585.1	NZ_CP053719.1	*Proteus mirabilis*	MPE0346	tRNA-PheU		
GCF_013358795.1	NZ_CP046048.1	*Proteus mirabilis*	HN2p			
GCF_014843115.1	NZ_CP062146.1	*Proteus mirabilis*	S012			
GCF_014931585.1	NZ_CP047929.1	*Proteus mirabilis*	ChSC1905			
GCF_015169015.1	NZ_CP063440.1	*Proteus mirabilis*	Yak_2019			
GCF_015693865.1	NZ_CP065147.1	*Proteus mirabilis*	PmBJ015-2			
GCF_015693965.1	NZ_CP065148.1	*Proteus mirabilis*	PmBJ012-2			
GCF_015693985.1	NZ_CP065144.1	*Proteus mirabilis*	PmBJ024-1			
GCF_015694005.1	NZ_CP065145.1	*Proteus mirabilis*	PmBJ023-2			
GCF_015694025.1	NZ_CP065146.1	*Proteus mirabilis*	PmBJ020-1			
GCF_016725905.1	NZ_CP068152.1	*Proteus mirabilis*	FDAARGOS_1079	tRNA-PheU		
GCF_016772335.1	NZ_CP044436.1	*Proteus mirabilis*	C55			
GCF_016772355.1	NZ_CP044437.1	*Proteus mirabilis*	C74			
GCF_016939715.1	NZ_CP070569.1	*Proteus mirabilis*	PM52260	tRNA-PheU		tRNA-PheV
GCF_016939735.1	NZ_CP070572.1	*Proteus mirabilis*	PM52808	tRNA-PheU		tRNA-PheV
GCF_017161055.1	NZ_CP046049.1	*Proteus mirabilis*	DY.F1.2			
GCF_017808555.1	NZ_CP066833.1	*Proteus mirabilis*	RGF134-1			
GCF_017901195.1	NZ_CP072779.1	*Proteus mirabilis*	1035			
GCF_018138945.1	NZ_CP073248.1	*Proteus mirabilis*	N292			
GCF_018138985.1	NZ_CP073246.1	*Proteus mirabilis*	N639-2X			
GCF_018139005.1	NZ_CP073247.1	*Proteus mirabilis*	S62-3-2-2			
GCF_018139105.1	NZ_CP073245.1	*Proteus mirabilis*	S74-3-2			
GCF_018336495.1	NZ_CP047589.1	*Proteus mirabilis*	SNYG35			
GCF_018972025.2	NZ_CP065039.2	*Proteus mirabilis*	XH1653			
GCF_019192645.1	NZ_CP077963.1	*Proteus mirabilis*	6Pmi283			
GCF_019443785.1	NZ_CP048692.1	*Proteus mirabilis*	HNS2p			
GCF_021228935.1	NZ_CP089317.1	*Proteus mirabilis*	PM1162	tRNA-PheV		
GCF_022353845.1	NZ_CP055009.1	*Proteus mirabilis*	STIN_74			
GCF_022354605.1	NZ_CP055095.1	*Proteus mirabilis*	SWHIN_109	tRNA-PheU		
GCF_022453625.1	NZ_CP092652.1	*Proteus mirabilis*	PM8762			
GCF_023093855.1	NZ_CP095765.1	*Proteus mirabilis*	T1010			
GCF_023242175.1	NZ_CP096775.1	*Proteus mirabilis*	HURS-181823	tRNA-PheV		tRNA-PheU
GCF_023242195.1	NZ_CP096776.1	*Proteus mirabilis*	HURS-186083	tRNA-PheV		tRNA-PheU
GCF_023702555.1	NZ_CP098446.1	*Proteus mirabilis*	FZP2826			
GCF_023702575.1	NZ_CP098447.1	*Proteus mirabilis*	FZP2936	tRNA-PheU		
GCF_023702595.1	NZ_CP098450.1	*Proteus mirabilis*	FZP3115	tRNA-PheU		
GCF_024138795.1	NZ_CP071773.1	*Proteus mirabilis*	swupm1			
GCF_024138815.1	NZ_CP071777.1	*Proteus mirabilis*	swupm2			
GCF_024138835.1	NZ_CP071780.1	*Proteus mirabilis*	swupm3			
GCF_025264285.1	NZ_CP031846.1	*Proteus mirabilis*	XH983	tRNA-PheV		
GCF_025398955.1	NZ_CP104698.1	*Proteus mirabilis*	NG-ABK-32			
GCF_025490355.1	NZ_CP104986.1	*Proteus mirabilis*	W47			
GCF_025998255.1	NZ_AP026827.1	*Proteus mirabilis*	NUITM-VP1			
GCF_026016045.1	NZ_CP110371.1	*Proteus mirabilis*	CZP17			
GCF_026016065.1	NZ_CP110372.1	*Proteus mirabilis*	CZP44			
GCF_026016085.1	NZ_CP110373.1	*Proteus mirabilis*	CZP26			
GCF_026016105.1	NZ_CP110376.1	*Proteus mirabilis*	NYP69			
GCF_026016125.1	NZ_CP110377.1	*Proteus mirabilis*	NYP73			
GCF_026016145.1	NZ_CP110375.1	*Proteus mirabilis*	NYP6			
GCF_026167565.1	NZ_CP110673.1	*Proteus mirabilis*	DP2019			
GCF_900635965.1	NZ_LR134205.1	*Proteus mirabilis*	NCTC4199	tRNA-PheV		
GCF_000754995.1	NZ_KN150745.1	*Proteus vulgaris*	ATCC_49132			
GCF_004116975.1	NZ_CP026364.1	*Proteus hauseri*	15H5D-4a			
GCF_022369495.1	NZ_CP059690.1	*Proteus penneri*	S178-2			

**Table 2:** Unique genes identified in *P. mirabilis* BL95, relative to *P. mirabilis* with complete genomes (Table 1). Some of these genes have paralogs in the genome of BL95 and other *Proteus* strains and may encode similar features or functions.

Locus_Tag	Genome Nucleotide Positions	Length	Direction	Product (GenBank Annotation)	Product (Prokka Annotation)	Genes *
QCK92_00170	29,331–29,576	246	reverse	AlpA family phage regulatory protein	hypothetical protein	
QCK92_00175	29,665–30,540	876	reverse	hypothetical protein	hypothetical protein	
QCK92_00180	30,633–31,898	1266	reverse	tyrosine-type recombinase/integrase	prophage integrase IntA	
QCK92_06810	1,482,955–1,483,173	219	reverse	HEAT repeat domain-containing protein	hypothetical protein	
QCK92_10410	2,229,236–2,229,460	225	reverse	type I toxin-antitoxin system ptaRNA1 family toxin	hypothetical protein	
QCK92_10415	2,229,524–2,229,772	249	reverse	hypothetical protein	hypothetical protein	
QCK92_10420	2,229,777–2,230,790	1014	reverse	P-type conjugative transfer protein TrbL	hypothetical protein	
QCK92_10425	2,230,694–2,231,203	510	reverse	type IV secretion system protein	hypothetical protein	
QCK92_10430	2,231,214–2,231,417	204	reverse	entry exclusion lipoprotein TrbK	not annotated	
QCK92_10435	2,231,461–2,232,240	780	reverse	P-type conjugative transfer protein TrbJ	hypothetical protein	
QCK92_10440	2,232,381–2,232,569	189	reverse	stabilization protein	hypothetical protein	
QCK92_10445	2,233,600–2,234,480	881	reverse	replication protein C, IncQ-type	hypothetical protein	
QCK92_10450	2,234,467–2,235,293	827	reverse	helicase RepA family protein	regulatory protein RepA	
QCK92_10455	2,235,298–2,235,519	222	reverse	AlpA family phage regulatory protein	hypothetical protein	
QCK92_10460	2,235,667–2,236,869	1203	reverse	tyrosine-type recombinase/integrase	prophage integrase IntA	
QCK92_12525	2,679,658–2,679,966	309	reverse	helix-turn-helix domain-containing protein	hypothetical protein	
QCK92_12530	2,680,018–2,680,206	189	reverse	DNA-binding protein	hypothetical protein	
QCK92_12535	2,680,233–2,680,589	357	forward	hypothetical protein	hypothetical protein	
QCK92_12540	2,680,907–2,681,245	339	forward	helix-turn-helix domain-containing protein	hypothetical protein	
QCK92_12545	2,681,381–2,682,151	771	reverse	DNA adenine methylase	hypothetical protein	
QCK92_12550	2,682,335–2,682,811	477	reverse	ABC transporter ATPase	hypothetical protein	
QCK92_12555	2,682,943–2,683,293	351	forward	putative holin	hypothetical protein	
QCK92_12645	2,695,862–2,696,143	282	forward	hypothetical protein	hypothetical protein	
QCK92_12700	2,704,416–2,705,507	1092	forward	phage tail protein	hypothetical protein	
QCK92_12705	2,705,507–2,706,286	780	forward	DUF4376 domain-containing protein	hypothetical protein	
QCK92_15815	3,431,156–3,431,878	723	reverse	4′-phosphopantetheinyl transferase superfamily protein	hypothetical protein	*HP*
QCK92_15820	3,432,045–3,433,091	1047	reverse	3-deoxy-7-phosphoheptulonate synthase	phospho-2-dehydro-3-deoxyheptonate aldolase, Trp-sensitive	*phzC*
QCK92_15825	3,433,123–3,434,535	1413	reverse	FAD-dependent oxidoreductase	hypothetical protein	*HP*
QCK92_15830	3,434,532–3,434,990	459	reverse	hypothetical protein	hypothetical protein	*HP*
QCK92_15835	3,435,058–3,436,827	1770	reverse	non-ribosomal peptide synthetase	dimodular nonribosomal peptide synthase	*dhbF*
QCK92_15840	3,436,876–3,437,802	927	reverse	hypothetical protein	hypothetical protein	*HP*
QCK92_15845	3,437,799–3,439,169	1371	reverse	aldehyde dehydrogenase family protein	hypothetical protein	*ehpG*
QCK92_15850	3,439,172–3,440,233	1062	reverse	AMP-binding protein	hypothetical protein	*ehpF*
QCK92_15855	3,440,246–3,440,881	636	reverse	pyridoxal 5′-phosphate synthase	phenazine biosynthesis protein PhzG	*phzG*
QCK92_15860	3,440,895–3,441,740	846	reverse	PhzF family phenazine biosynthesis protein	trans-2,3-dihydro-3-hydroxyanthranilate isomerase	*phzF*
QCK92_15865	3,441,719–3,442,072	354	reverse	hypothetical protein	hypothetical protein	*HP*
QCK92_15870	3,442,069–3,443,934	1866	reverse	anthranilate synthase family protein	isochorismate synthase MenF	*phzE*
QCK92_15875	3,443,931–3,444,551	621	reverse	isochorismatase family protein	Phenazine biosynthesis protein PhzD	*phzD*
QCK92_15880	3,444,627–3,445,085	459	reverse	PhzA/PhzB family protein	phenazine biosynthesis protein PhzB	*phzA/B*
QCK92_15885	3,445,132–3,445,914	783	reverse	SDR family NAD(P)-dependent oxidoreductase	3-oxoacyl-[acyl-carrier-protein] reductase FabG	*ehpK, fabG*
QCK92_15890	3,446,019–3,446,405	387	reverse	VOC family protein	phenazine antibiotic resistance protein EhpR	*ehpR*
QCK92_15895	3,446,919–3,447,617	699	forward	DUF2461 domain-containing protein	hypothetical protein	*HP*
QCK92_15905	3,448,489–3,449,697	1209	forward	IS256 family transposase	IS256 family transposase ISEic2	*tnp_IS256*
QCK92_15910	3,449,845–3,450,042	198	reverse	hypothetical protein	not annotated	*HP*
QCK92_15915	3,450,152–3,450,505	354	reverse	hypothetical protein	hypothetical protein	*cdiI-o2*
QCK92_15920	3,450,518–3,451,501	984	reverse	cysteine peptidase family C39 domain-containing protein	hypothetical protein	*cdiA-o2*
QCK92_15925	3,451,663–3,452,433	771	reverse	VENN motif pre-toxin domain-containing protein	deoxyribonuclease CdiA	*cdiA-o2′*
QCK92_15930	3,452,688–3,453,185	498	reverse	contact-dependent growth inhibition system immunity protein	immunity protein CdiI-o11	*cdiI-o1*
QCK92_15935	3,453,316–3,453,561	246	reverse	hypothetical protein	deoxyribonuclease CdiA-o11	*cdiA-o1*
QCK92_15940	3,453,536–3,453,922	387	reverse	hypothetical protein	deoxyribonuclease CdiA-o11	*cdiA-o1′*
QCK92_15945	3,454,113–3,454,409	297	forward	SymE family type I addiction module toxin	not annotated	*symE*
QCK92_15950	3,454,471–3,454,740	270	reverse	hypothetical protein	hypothetical protein	*HP*
QCK92_15955	3,454,771–3,455,304	534	reverse	contact-dependent growth inhibition system immunity protein	immunity protein CdiI-YPIII	*cdiI*
QCK92_15960	3,455,502–3,455,945	444	reverse	hypothetical protein	hypothetical protein	*HP*
QCK92_15965	3,455,942–3,465,757	9816	reverse	polymorphic toxin type 25 domain-containing protein	tRNA nuclease CdiA	*cdiA*
QCK92_16130	3,495,436–3,495,606	171	forward	hypothetical protein	hypothetical protein	

* Shown are gene names as used in this work.

**Table 3:** Genes and encoded proteins in the phenazine biosynthesis operon of *P. mirabilis* BL95. Different functional gene categories are indicated as follows: A, core phenazine biosynthesis; B, modification of phenazine trycycle; C; resistance to phenazines. For additional/alternative protein names, see Table 2.

Locus_Tag	Protein Name	Commonly Used Gene Name	Gene Length	Functional Category
QCK92_15815	4′-phosphopantetheinyl transferase superfamily protein		723	B
QCK92_15820	3-deoxy-7-phosphoheptulonate synthase	*phzC, aroH*	1047	A
QCK92_15825	FAD-dependent oxidoreductase		1413	B
QCK92_15830	hypothetical protein		459	B
QCK92_15835	non-ribosomal peptide synthetase	*dhbF*	1770	B
QCK92_15840	hypothetical protein		927	B
QCK92_15845	aldehyde dehydrogenase family protein	*ehpG*	1371	B
QCK92_15850	AMP-binding protein	*ehpF*	1062	B
QCK92_15855	pyridoxal 5′-phosphate synthase	*phzG*	636	A
QCK92_15860	PhzF family phenazine biosynthesis protein	*phzF*	846	A
QCK92_15865	hypothetical protein *		354	A
QCK92_15870	anthranilate synthase family protein	*phzE, menF2*	1866	A
QCK92_15875	isochorismatase family protein	*phzD*	621	A
QCK92_15880	PhzA/PhzB family protein	*phzA/B*	459	A
QCK92_15885	SDR family NAD(P)-dependent oxidoreductase	*ehpK, fabG*	783	B
QCK92_15890	VOC family protein	*ehpR*	387	C

* Although the function of this protein is currently unknown, the presence of its homologs in diverse phenazine biosynthesis systems suggests that it may belong to the core phenazine biosynthesis enzymes.

## Data Availability

The genome sequence data of *P. mirabilis* BL95 were deposited in NCBI GenBank under BioProject accession number PRJNA954452, BioSample accession number SAMN34145880, and assembled chromosome CP122400.

## Supplementary Materials

The following supporting information can be downloaded at: https://www.mdpi.com/article/10.3390/microorganisms12071321/s1, Figure S1: Pangenome of *P. mirabilis*. Overall, the pangenome consists of 11,364 genes identified in 99 *P. mirabilis* strains; Figure S2: Schematic representation of the *P. mirabilis* BL95 chromosome using Proksee (https://proksee.ca, accessed on 16 December 2022); Figure S3: Similarity of the CDI region from ICE*Pm2* in *P. mirabilis* to homologous regions from other species; Table S1: Genetic organization of Integrative and Conjugative Elements ICE*Pm1*, ICE*Pm2*, and ICE*Pm3* shown in Figure 5; Table S2: Genes in *P. mirabilis* BL95 corresponding to secretion systems in gram-negative bacteria.
