# Supplementary material for: Pangenome Analysis Reveals Novel Contact-Dependent Growth Inhibition System and Phenazine Biosynthesis Operons in Proteus mirabilis BL95 That Are Located in An Integrative and Conjugative Element
Source: Microorganisms. 2024 Jun 28;12(7):1321. doi: 10.3390/microorganisms12071321 (PMC11278526; doi:10.3390/microorganisms12071321)
Supplement: Supplementary file 1 [file microorganisms-12-01321-s001.zip › FiguresS1-S3.pdf]

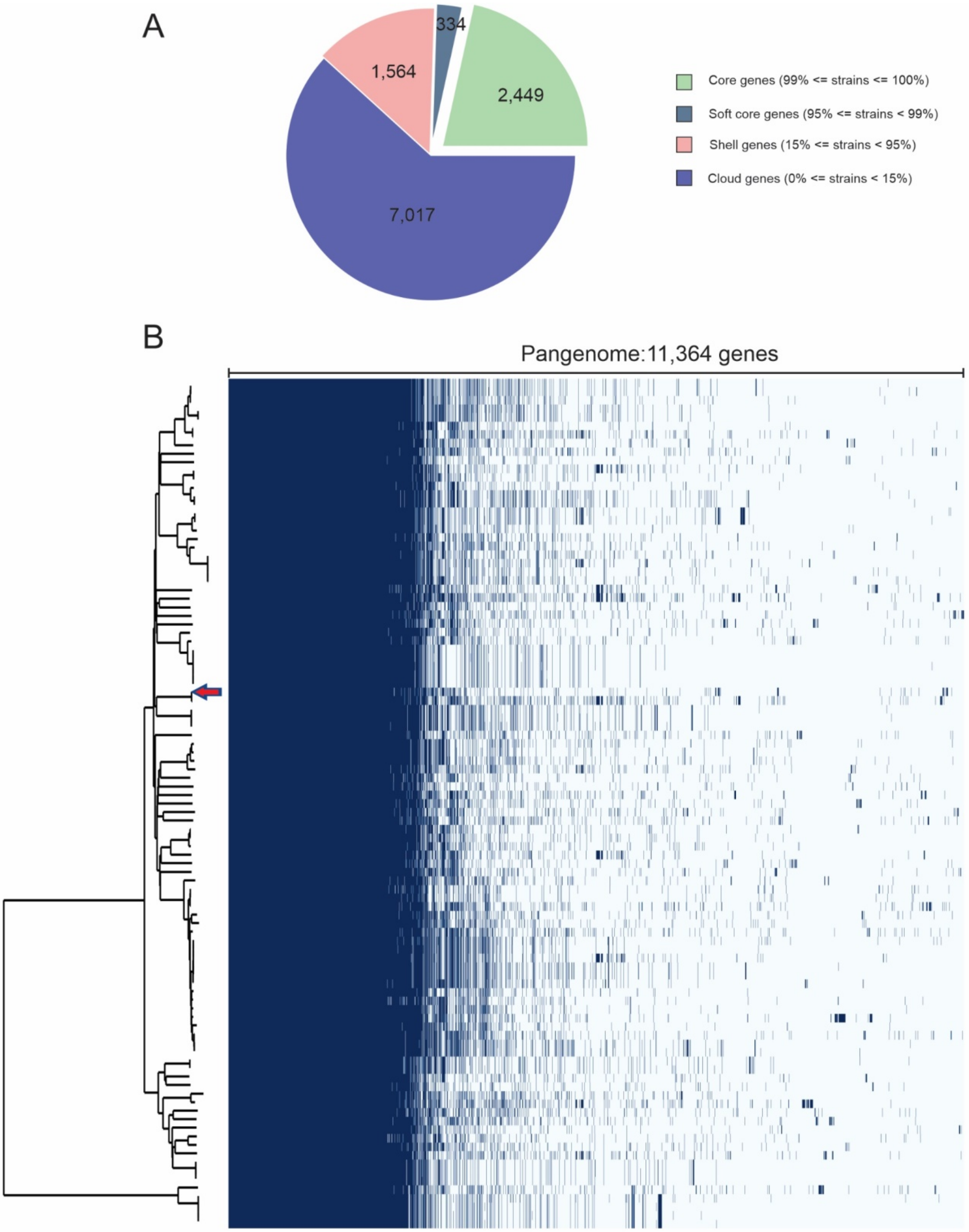

Figure S1. Pangenome of *Proteus mirabilis*. Overall, pangenome consists of 11,364 genes identified in 99 *P. mirabilis* strains. A. Pie chart shows distribution and classification of genes according to their occurrence among strains. B. Phylogeny of *P. mirabilis* combined with heatmap showing presence (blue) or absence (white) of genes in the studied strains. The phylogeny, shown on the left, was constructed using 2,449 core genome genes (see Figure 1). Red arrow indicates position of BL95.

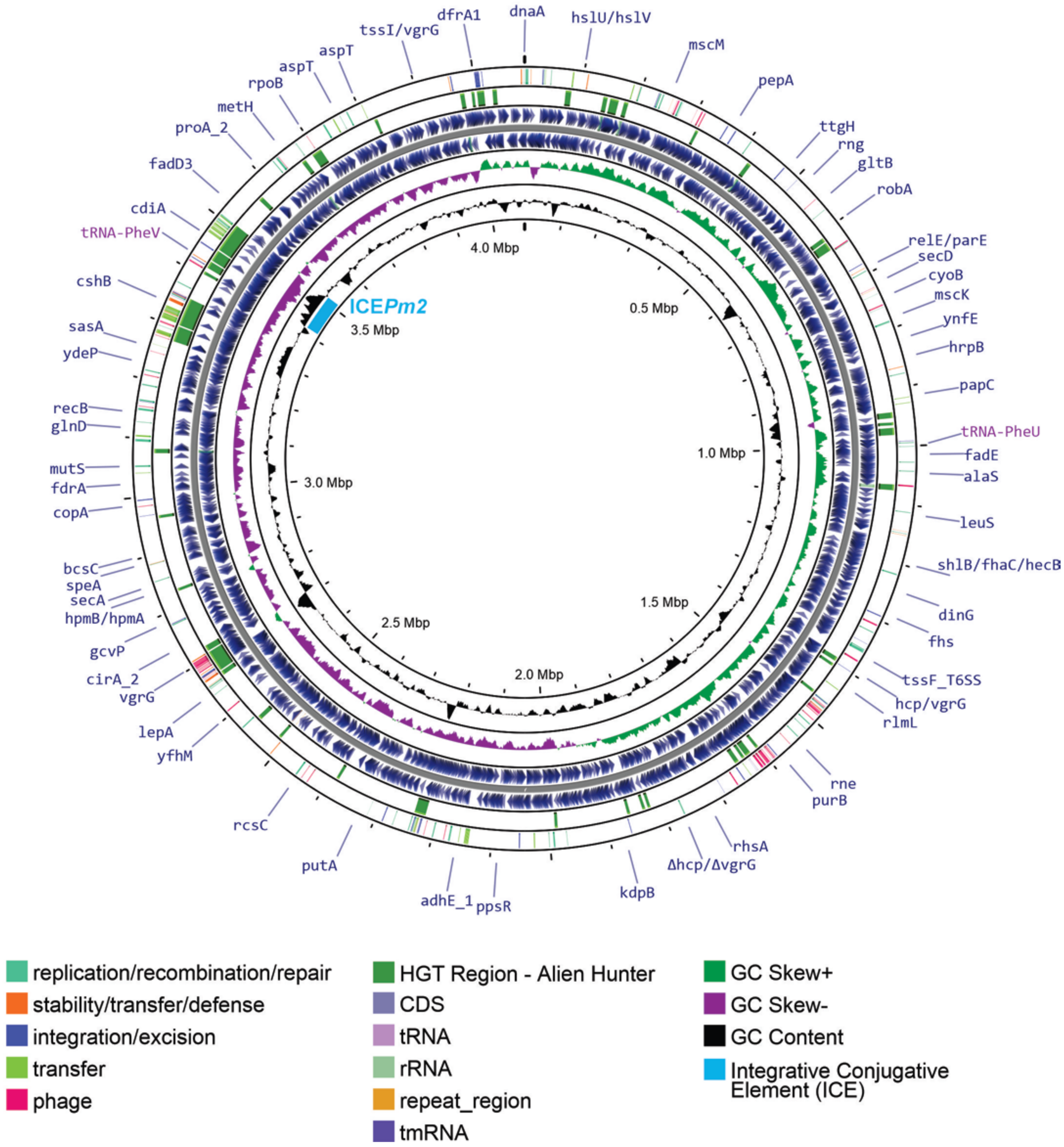

Figure S2. Schematic representation of the *P. mirabilis* BL95 chromosome using Proksee (https://proksee.ca). The scale is shown in megabases on the black innermost circle. Moving inward, Ring 1 shows genes according to their functional category. Ring 2 shows regions resulting from horizontal gene transfer, as identified by Alien Hunter. Ring 3 and Ring 4 show forward and reverse strand CDSs, respectively, in dark blue. Also shown on these rings are rRNAs (light green arrows) and tRNAs (light purple arrows). Ring 5 shows GC skew and Ring 6 shows GC content. Blue arc shown above the innermost circle indicates ICE*Pm2*.

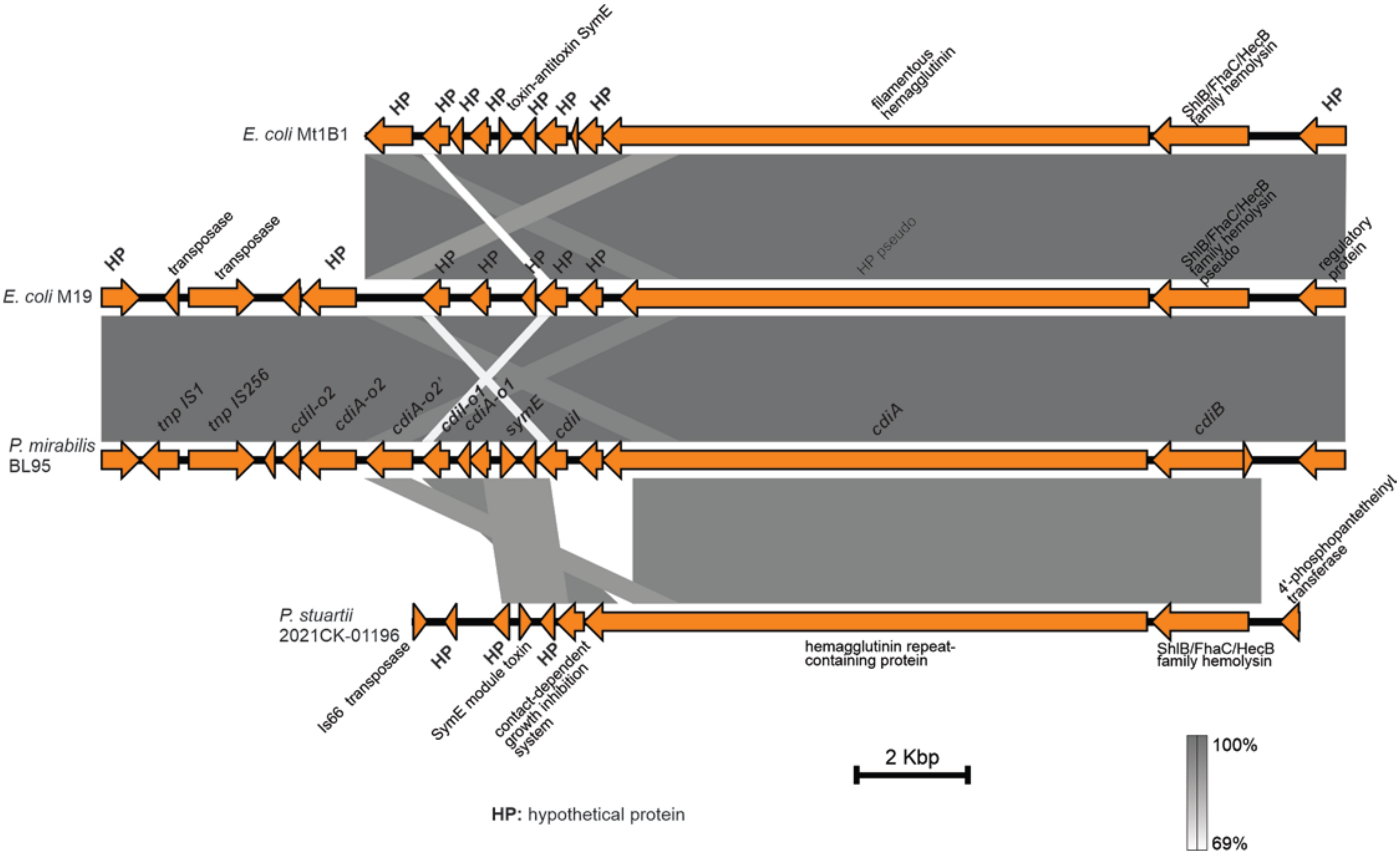

Figure S3. Similarity of the CDI region from ICE*Pm2* in *P. mirabilis* to homologous regions from other species. Gene names are shown for *P. mirabilis* BL95. Probable orphan genes are indicated by suffix '-o'. Original annotations (as in GenBank Accessions) are shown for other species, illustrating difficulties annotating two-partner systems such as CDI. Gray rectangles outline regions of homology, and their shades show sequence similarity ac-cording to the vertical scale bar. Comparison is done using EasyFig [28].
